# Perirhinal cortical inactivation impairs object-in-place memory and disrupts task-dependent firing in hippocampal CA1, but not in CA3

**DOI:** 10.3389/fncir.2013.00134

**Published:** 2013-08-14

**Authors:** Inah Lee, Seong-Beom Park

**Affiliations:** Department of Brain and Cognitive Sciences, Seoul National UniversitySeoul, South Korea

**Keywords:** object-place memory, episodic memory, event memory, hippocampus, perirhinal cortex, CA1, CA3

## Abstract

Objects and their locations can associatively define an event and a conjoint representation of object-place can form an event memory. Remembering how to respond to a certain object in a spatial context is dependent on both hippocampus and perirhinal cortex (PER). However, the relative functional contributions of the two regions are largely unknown in object-place associative memory. We investigated the PER influence on hippocampal firing in a goal-directed object-place memory task by comparing the firing patterns of CA1 and CA3 of the dorsal hippocampus between conditions of PER muscimol inactivation and vehicle control infusions. Rats were required to choose one of the two objects in a specific spatial context (regardless of the object positions in the context), which was shown to be dependent on both hippocampus and PER. Inactivation of PER with muscimol (MUS) severely disrupted performance of well-trained rats, resulting in response bias (i.e., choosing any object on a particular side). MUS did not significantly alter the baseline firing rates of hippocampal neurons. We measured the similarity in firing patterns between two trial conditions in which the same target objects were chosen on opposite sides within the same arm [object-in-place (O-P) strategy] and compared the results with the similarity in firing between two trial conditions in which the rat chose any object encountered on a particular side [response-in-place (R-P) strategy]. We found that the similarity in firing patterns for O-P trials was significantly reduced with MUS compared to control conditions (CTs). Importantly, this was largely because MUS injections affected the O-P firing patterns in CA1 neurons, but not in CA3. The results suggest that PER is critical for goal-directed organization of object-place associative memory in the hippocampus presumably by influencing how object information is associated with spatial information in CA1 according to task demand.

## Introduction

A large body of literature suggests that the hippocampus is important for remembering objects and their associated locations (Parkinson et al., 1988; Cahusac et al., 1989; Save et al., 1992; Gilbert and Kesner, 2004; Lee and Solivan, 2008; Manns and Eichenbaum, 2009; Kim et al., 2011; Yoon et al., 2012), which presumably subserves episodic memory (Tulving and Markowitsch, 1998). It is hypothesized that the hippocampus receives critical information from the cortical areas in the medial temporal lobe such as PER, postrhinal cortex (POR), lateral entorhinal cortex (LEC), and medial entorhinal cortex (MEC; Witter and Amaral, 2004). However, details as to which of these extrahippocampal regions process which aspects of object-place associative memory still remain largely unknown. A currently dominant theory posits that the PER-to-LEC stream processes non-spatial information such as object identity, whereas spatial information is computed and transmitted through the POR-to-MEC stream before the two streams merge in the hippocampus (Hargreaves et al., 2005; Knierim et al., 2006; Kerr et al., 2007; Lee and Lee, 2013). In the current study, we tested the effects of inactivating PER on task-related firing patterns of hippocampal subfields CA1 and CA3 in an object-place memory task in rats.

The object-place association task in the current study was repeatedly used in our prior studies and we have demonstrated its dependence on the dorsal hippocampus, PER, and the medial prefrontal cortex (mPFC; Lee and Solivan, 2008, 2010; Lee and Kim, 2010; Jo and Lee, 2010a,b; Kim et al., 2011). The task required the rats to visit one of the two arms in a radial maze and choose a particular object that was rewarded in a given arm. For example, the rat should displace object-A (but not object-B) in arm 3 and should choose object-B (but not object-A) in arm 5 irrespective of the within-arm object positions in order to obtain reward. Initially before learning takes place, rats typically adopt a response strategy (choosing any object on the left side, for example), or *response-in-place (R-P)* strategy, instead of paying attention to the identity of the object being chosen. However, as learning progresses, the rat drops the response strategy and starts to choose the target object in association with arm information regardless of the within-arm locations of the object. We call this an *object-in-place (O-P)* strategy. We showed previously that, prior to learning, the spatial firing rate maps of neurons in the dorsal CA1 of the hippocampus (and mPFC) were highly correlated between the trial conditions in which R-P strategy was used. However, after learning took place, the firing rate maps of neurons became uncorrelated in R-P trials, but were highly correlated between the trial conditions in which O-P strategy was used (Lee and Kim, 2010; Kim et al., 2011). This suggests that object information became represented in the hippocampus in this task as learning took place.

In order to test the source of the object information, in the current study, we tested the contributions of PER to the strategy-dependent firing patterns of hippocampal neurons. If object information available in the hippocampus is critically dependent on PER, inactivation of PER should disrupt the O-P firing patterns normally observed in the hippocampus after learning. More important, we examined whether different subfields of the hippocampus exhibited heterogeneous responses to the PER inactivations.

## Materials and methods

### Subjects

Three male Long–Evans rats (300–400 g) were used in the current study. Food was controlled for maintaining their body weights at around 85% of free-feeding weights but water was available *ad libitum*. Twelve-hour light-dark cycle was used. All the protocols for animal care and surgery followed the guidelines of the National Institute of Health and the Institutional Animal Care and Use Committee.

### Object-place paired-associate task

**Figure 1:**
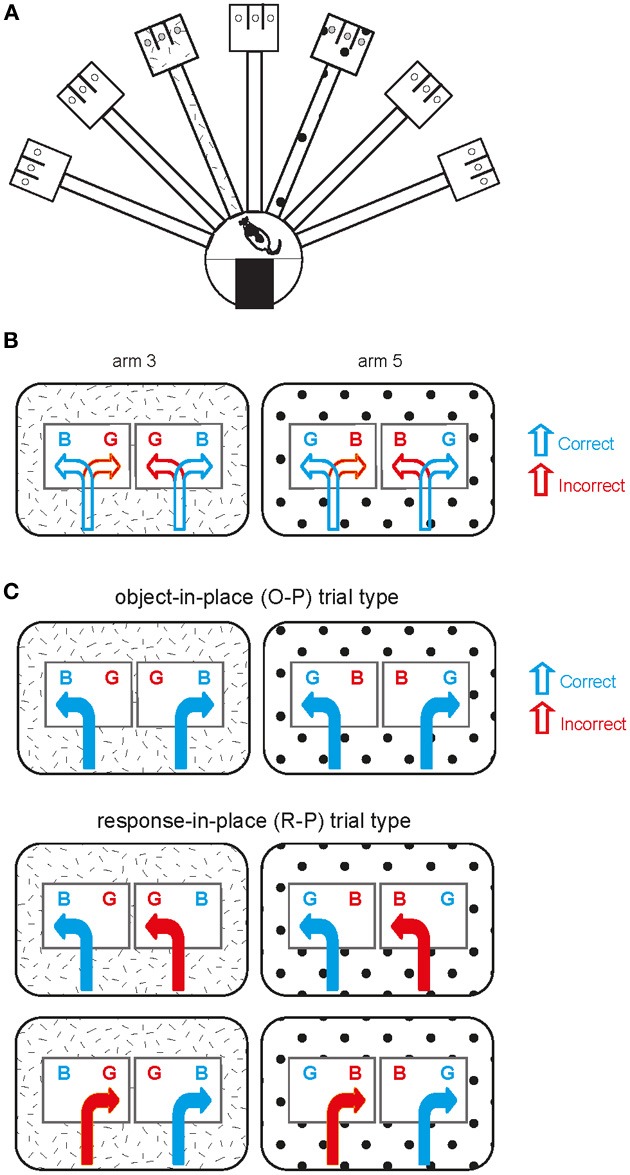
**Object-place paired association task. (A)** Illustration of an overview of the radial arm maze used in the current study. The hatched pattern and polka-dot pattern in arm 3 and arm 5 are for illustration purposes only (see below) and there was no explicit local cue in the real maze. **(B)** Schematic illustration of the behavioral task. Two arms are represented by the patterns (distinct spatial contexts) in the outer rectangular areas. Two possible object configurations (BG and GB with B standing for obj-B and G for obj-G; B and G were rewarded in arm 3 and arm 5, respectively) are shown with two possible behavioral choices (correct and incorrect choices) per object configuration in each arm. **(C)** Illustration of different trial types observed within a session. Object-in-place (O-P) trial type includes the trials in which opposite response directions are associated with choosing the same target object in a given arm, whereas response-in-place (R-P) trial type involves the trials in which the same directional responses to different objects lead to a correct choice in one case but to an incorrect choice in the other case in a given arm.

Detailed descriptions of the behavioral apparatus and the task can be found in our previous studies (Lee and Solivan, 2008, 2010; Jo and Lee, 2010a,b; Lee and Kim, 2010; Kim et al., 2011). Briefly, a radial-arm maze in a circular curtained area was used in the study (Figure 1A). Only two arms (arm 3 and arm 5) of seven arms in the maze were used and those two arms were associated with distinct visual contexts (i.e., distal cues along the curtains) in the room. Once entering an open arm (only one arm being opened per trial) and approaching the choice platform (rectangular platform at the end of the arm), the rat was required to displace one of the toy objects (toy figure—Barney and Girl, each abbreviated as obj-B and obj-G henceforth; Figure 1B) in order to retrieve a piece of cereal reward in the food-well underneath the object. Rats should choose obj-B in arm 3 regardless of its position within the choice platform and obj-G in arm 5 (thus biconditional object-place paired association; Figure 1B). Sixty-four trials were given in a recording session (1 session per day) and equal numbers of both arm conditions were presented during the session. Rats were trained to criterion (75% or higher performance in each arm) for 10 days on average before receiving surgeries for hyperdrive implantations. The rats were continuously trained after surgery while tetrodes were moved to target locations across many days (range: 7–13 days).

### Hyperdrive and cannula-implantation surgery

**Figure 2:**
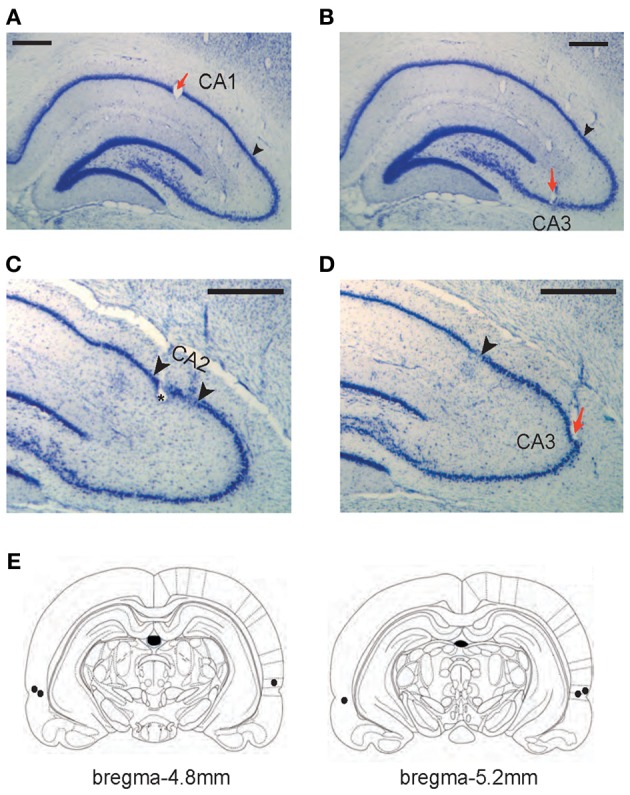
**Histological verification of recording and drug-injection locations. (A–D)** Representative tetrode locations for CA1 **(A)**, CA3 (**B** and **D)**, and CA2 **(C)**. The tetrodes whose final recording positions were identified in CA2 were not included in final analyses. Red arrows indicate tetrode-tip locations and black arrowheads in A, B, and D denote the proximal end of CA1 layer (CA1-CA2 border). Two arrowheads in C mark putative CA2 boundaries (~300 μm from the CA1-CA2 boundary). Scale bars = 500 μm. **(E)** Illustration of the injection cannula-tip positions (black dots) in PER for three rats. Each section's longitudinal position in reference to brema is given.

Each rat was implanted with a hyperdrive with 16 tetrodes plus 2 reference electrodes in the dorsal hippocampus once the rat satisfied the performance criterion for two consecutive days. In addition, bilateral cannulae in PER were implanted at the same time. Detailed descriptions of the surgical procedures can be found elsewhere (Lee and Kim, 2010; Jo and Lee, 2010a; Kim et al., 2011) and only briefly explained here. For the hyperdrive implantation, nichrome wires were used to construct tetrodes and the impedance of each tetrode was set in the range of 150–300 kΩ at 1 kHz. The tetrode bundle targeted the dorsal hippocampus in the right hemisphere. Among the 16 tetrodes in a given bundle, 6 and 10 tetrodes targeted CA1 (Figure 2A) and CA3 (Figures 2B,D) cell layers, respectively.

Bilateral cannulae (26G guide cannulae coupled with 32G stylets) were also implanted for targeting PER (Figure 2E) in the same animal following the procedures described elsewhere (Jo and Lee, 2010a). Briefly, before a hyperdrive was implanted, small burr holes were drilled in the parietal bone with the following coordinates: 4.8 mm posterior to bregma, 7.6 mm lateral to midline at an angle of 10° with the tip oriented medially, 3.9 mm ventral from the skull surface. The cannulae were secured in place with bone cement over skull screws.

### Electrophysiological recording

Detailed descriptions of the electrophysiological recording setups and recording procedures were given in our previous studies (Lee and Kim, 2010; Kim et al., 2011). Briefly, tetrodes were adjusted while the rat slept in a recording booth outside the behavioral recording room. Signals from spiking channels were amplified 1,000–10,000 times and digitized at 32 kHz sampling rate (filtered at 300–6,000 Hz) by using a Digital Lynx data acquisition system (Neuralynx, Bozeman, MT). During behavioral recording sessions, neural signals were fed through a slip-ring commutator. The animal's position data were captured by detecting LED lights in the head stage through a digital camera on the ceiling and fed to a frame grabber at 30 Hz sampling rate in the data acquisition PC to be processed together with neural signals.

### Intracranial microinjections

Once ready for behavioral testing after recovery from surgery, the rat was first run without any drug injection until showing criterion performance (≥75% for each arm for 2 days consecutively). Then, vehicle solution (sterile physiological saline) was injected 30 min before behavioral testing. Muscimol was injected during the next 2 days of testing followed by another vehicle injection on the last day. Detailed procedures for drug injection in PER can be found in our prior studies (Jo and Lee, 2010a). Briefly, MUS (0.5 μg/0.5 μL) or sterile physiological saline (SAL) was injected at 10 μL/h rate bilaterally using a Hamilton syringe and a microinjection pump while the rat was under light gas anesthesia by isoflurane. The injection cannula was left in place for an additional 1 min to achieve a proper diffusion of the drug from its tip. The rat was returned to its home cage and the behavioral recording commenced in 30 min.

### Histology and reconstruction of tetrode tracks

The tetrode tip locations and cannula tip positions were assessed histologically following the procedures previously described (Jo and Lee, 2010a; Kim et al., 2011). After the completion of all experiments, marker lesions (10 μA current for 10 s) were made on all tetrode tips 24 h before perfusion. Then, each rat was transcardially perfused by 0.9% SAL and 10% formaldehyde solution after receiving a lethal dose of pentobarbital. The decapitated head of the rat was stored in the formalin solution for 24 h additionally to ensure an easy identification of individual tetrode tracks. When the brain was extracted, the tetrode bundle was examined under the microscope as soon as the skull was detached from the head in order to verify that all tetrodes came out of the bundle straight and in parallel with each other. We verified that all tetrodes came out straight without crossing the tracks of the adjacent tetrodes. The brain was frozen and cut in coronal sections (40 μm) and the sections were Nissl-stained with thionin.

All hippocampal sections were then photomicrographed digitally into graphical files in bitmap format. Once digitized, the tetrode tracks and the principal cell layers were digitally traced in Photoshop (Adobe, San Hose, CA) using a pen tablet. At the time of tracing, the borders between the CA1 and CA2 were marked. The CA1-CA2 border was easily identifiable in Nissl sections (and with the help of highly magnified images under the microscope) because of a sudden widening of the cell layer at the junction between the proximal CA1 and distal CA2 (Amaral and Lavenex, 2007; Henriksen et al., 2010) (Figures 2C,D). Once digitally traced, the serial 2D images of tetrode tracks and cell layers were three-dimensionally reconstructed using commercial software (Voxwin, Voxar, UK). The resulting 3D image (i.e., the tetrodes as a bundle and the relative positions of hippocampal cell layers) was rotated to identify the pre-surgical configuration of the tetrode bundle. With the help of this 3D-reconstructive approach with the CA1-CA2 border being also reconstructed with the electrode tracks (Lee et al., 2004a; Henriksen et al., 2010), it was possible to assign individual tetrodes to either CA1 or CA2-CA3 subfields. It was very difficult, however, to identify the exact borders between CA2 and CA3 in Nissl-stained sections and we excluded 4 tetrodes that recorded neurons from putative CA2 (within ~300 μm from the CA1-CA2 boundary; Figure 2C) in CA3 analyses. Excluding additional 5 tetrodes that were located within ~500 μm from the CA1-CA2 boundary, however, did not produce any difference in the main results of the study. Most CA3 tetrodes used in final analyses were located in the CA3b-c region (Lorente de No, 1934) (Figures 2B–D) and all CA1 recordings were made in the proximal CA1 region.

### Data analysis

Unit isolation and other neural data analyses were conducted as described elsewhere (Lee et al., 2004a,b; Hargreaves et al., 2005; Lee and Kim, 2010; Kim et al., 2011). Spikes from single units were isolated off-line using Windows-based custom software. Multiple waveform parameters such as peak, width, height, and energy associated with four wires of a tetrode were compared for unit isolation. Interspike interval histograms were also examined for ensuring single-unit activity. Only putative complex spike cells were used (based on firing rate and spike width) and putative theta cells were not used. The isolated units were used in final analyses only if the number of spikes fired in one of the arms exceeded 50 (average firing rate ≥1 Hz) and when the spatial information score exceeded 0.5 (Lee and Kim, 2010; Kim et al., 2011). Furthermore, the units (i.e., clusters) that were used in final analyses should satisfy the additional criteria of isolation distance ≥12 and L-ratio ≤0.2 (Harris et al., 2001; Schmitzer-Torbert et al., 2005). Spatial information score (Skaggs et al., 1993) was calculated with the following formula.

$$\text{Spatial information (bit/spike)}=\int \lambda (x)\log_{2}\frac{\lambda (x)}{\lambda }p(x)dx,$$

where *x* is spatial position, *p*(*x*) is the probability density for being at position *x*, λ (*x*) is the mean firing rate when the rat is at position *x*, and λ is the overall mean firing rate of the unit. For spatial information, only statistically significant scores were considered (*p* < 0.001).

The amount of similarity between different trial conditions was measured by calculating a Pearson correlation coefficient in this study (Lee and Kim, 2010; Kim et al., 2011). First, the recording area was coded as a 72 × 48 matrix for constructing a two-dimensional spatial firing rate map. The 2D rate map for a single unit was constructed by dividing the number of spikes generated by a neuron in a given bin by the duration of stay in seconds in that bin. An outbound journey was defined as the period from the moment the rat entered the arm until one of the objects was pushed in the choice platform. An inbound journey was defined as the period from the end of the outbound journey to exiting the arm to enter the start box. Only outbound journeys were used for analysis in this study. The trials in which the rat chose the same target object on either side of the choice platform within a given arm were called O-P trials and the trials in which the rat chose any object encountered on a particular side of the choice platform within the same arm were named R-P trials. For the rate maps constructed from the O-P trials, there were two types: one being composed of trials in which the rat chose the object on the left side and the other type being composed of trials in which the target object was on the right side. The rate maps constructed in associations with these two trial conditions were cross-correlated to obtain a Pearson correlation coefficient (O-P trial type in Figure 1C). The trials belonging to O-P trial type include only correct trials. Only the arm areas were used in the correlation analysis because the position traces significantly diverged once entering the choice platform between the two conditions (Lee and Kim, 2010; Kim et al., 2011). Pearson's correlation was calculated only using the pixels overlapping between the two trial types. The same methods were applied for calculating the rate-map similarity between R-P trial conditions (R-P trial type in Figure 1C). Pearson's correlation coefficients were calculated between correct and incorrect trials sharing the same turning direction (but different objects) in R-P trial type. Once a correlation coefficient (*r*) was calculated, Fisher's r-to-z transformation was performed on the correlation coefficient in order to make the format of the results comparable to the results from our previous studies (Lee and Kim, 2010; Kim et al., 2011). The following formula was used:

$$z_{r}=\frac{1}{2}\left[\log(1+r)-\log(1-r)\right]$$

The resulting z-transformed correlation coefficients of single units were presented in the form of a histogram in the study, and comparisons of the distributions from different conditions were performed using a Kolmogorov–Smirnov test. Skewness of a distribution is measured by calculating the ratio of the third moment of the distribution divided by the cube of standard deviation (Kirk, 1989).

For both behavioral and neural data analyses, we combined the 2 days of no-drug injection conditions and 2 days of SAL injections (pre- and post-MUS SAL conditions) into one control condition (CT) and the 2 days of MUS conditions into a single inactivation condition (MUS) to increase statistical power. No significant differences were found in behavioral performance between no-drug condition and pre-MUS SAL condition [*t*_(2)_ = 1.01, *p* > 0.1, paired *t*-test], between no-drug condition and post-MUS SAL condition [*t*_(2)_ = 1.29, *p* > 0.1], and between the pre- and post-MUS SAL conditions [*t*_(2)_ = 4.21, *p* > 0.05]. No significant difference was found when the average performance of no-drug condition was compared with the average performance in SAL condition [pre- and post-MUS SAL conditions combined; *t*_(2)_ = 0.19, *p* > 0.5; paired *t*-test]. Also, there was no significant difference in performance between the two MUS conditions [*t*_(2)_ = 1.41, *p* > 0.1; paired *t*-test]. A response-bias index was calculated by obtaining the absolute value of the difference between the number of left choices and the number of right choices, divided by the sum of the two.

## Results

### Histological verification of tetrode- and cannula-tip positions

All the tetrodes included in data analyses were placed in the dorsal hippocampus (Figures 2A–D). All CA1 tetrodes were in the proximal CA1 region (closer to CA3) and CA3 tetrodes were distributed along the proximal-to-distal axis (from CA3a to CA3c). Several tetrodes were located in the hilar CA region (between the lower and upper blades of the dentate gyrus) and these were not used in data analyses because it was difficult to pinpoint the recorded cell layer on a given day in this region. The bilateral cannula tips (injection cannula tips) were located in the PER in all three rats (Figure 2E). According to the rat atlas (Paxinos and Watson, 1986), the PER runs ~4 mm longitudinally (between 3 and 7 mm posterior to bregma) and we targeted the mid-PER (~5 mm posterior to bregma) along the anterior-posterior axis. We have demonstrated in our previous study that the implantation coordinates and drug-injection parameters used in the current study resulted in a fairly localized spread of fluorescent MUS in PER both anteroposteriorly and mediolaterally although the most extreme anterior and posterior parts of PER were spared (Jo and Lee, 2010a).

### Inactivation of PER severely disrupted performance in the object-place association task

**Figure 3:**
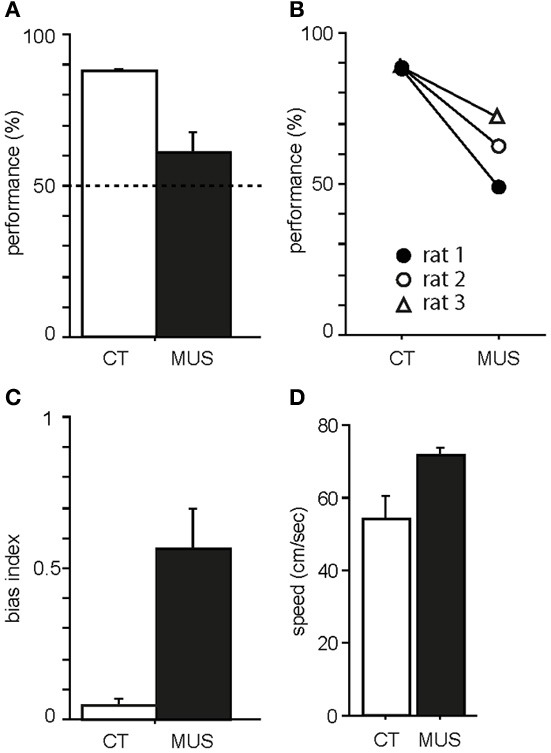
**Behavioral performance in the task. (A)** Average percent correct scores in CT and MUS conditions. Dotted line indicates chance level. Note the significant impairment in performance in MUS compared to CT. Mean ± s.e.m. **(B)** Performances of individual rats. Note that all rats showed the same trend of decrease in performance under MUS. **(C)** Response bias in MUS condition compared to CT condition. Mean ± s.e.m. **(D)** Average moving speed of drug groups during the task. Mean ± s.e.m.

As previously shown in our studies (Jo and Lee, 2010a,b), PER-MUS markedly impaired performance in the task. Specifically, rats showed almost 90% correct performance in the CT condition (Figure 3A). The MUS injections markedly impaired performance compared to the vehicle conditions and a paired *t*-test showed a significant drug effect on performance [*t*_(2)_ = −4.64, *p* < 0.05]. The performance drops with MUS were observed in all three animals (Figure 3B) so the behavioral deficits did not originate from only a subset of subjects. Animal behavior was governed by the R-P strategy when MUS was injected in PER, resulting in high response bias in the response-bias index [*t*_(2) = −4.72,_ *p* < 0.05; Figure 3C]. No significant difference was found in the animal's moving speed between the CT and MUS conditions [Figure 3D; *t*_(2)_ = −3.79, *p* > 0.05; paired *t*-test]. These results along with the results from our previous studies (Lee and Solivan, 2008, 2010; Jo and Lee, 2010a,b) strongly suggest that the current task is critically dependent on the normal operations of the dorsal hippocampus and PER.

### Inactivation of PER did not alter the basic firing properties of hippocampal neurons

**Figure 4:**
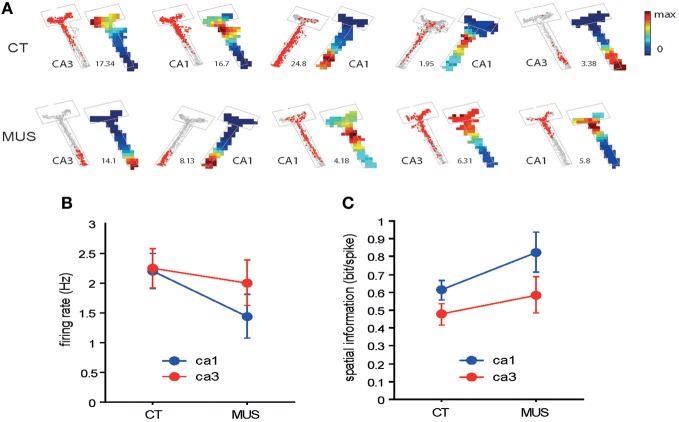
**Overall spatial firing of CA1 and CA3 neurons. (A)** Examples of spatial firing patterns of individual neurons from CA1 and CA3 in different drug conditions. For each neuron, a raw spiking map (gray dot = position trace) is presented on the left and the associated firing rate map is shown on the right. The number between the two indicates maximal firing rate (Hz). The boundary of the arm is also shown in gray lines. Firing rates are color-scaled from 0 to maximum for each neuron. The neurons presented were not simultaneously recorded. **(B)** Overall firing rates of neurons in CA1 and CA3 in different drug conditions. No statistically significant differences were found between the two conditions. Mean ± s.e.m. **(C)** Comparison of spatial information in CA1 and CA3 neuronal firing between the two drug conditions. No statistical significance was observed between the two drug conditions. Mean ± s.e.m.

As numbers of cells recorded from CA1 and CA3 in different drug conditions are shown in Table 1, all rats contributed unit data to CA1 and CA3 analyses in the study. We first examined whether MUS significantly altered the basic firing properties of principal neurons in the hippocampus during the task. The overall spatial firing patterns of CA1 and CA3 units on the maze looked similar between CT and MUS conditions and no obvious differences were noticed based on the firing rate maps (Figure 4A). A Two-Way ANOVA showed no significant effects of drug [*F*_(1, 187)_ = 1.67, *p* > 0.1], subfield [*F*_(1, 187)_ = 0.59, *p* > 0.1], and the interaction between drug and subfield [*F*_(1, 187)_ = 0.44, *p* > 0.5] on the average firing rate (Figure 4B).

**Table 1:** **The number of units in CA1 and CA3 used in final analysis across different drug conditions in individual rats**.

	**CT**						**MUS**			**Total**
	**No drug**			**SAL**						
	**Rat 1**	**Rat 2**	**Rat 3**	**Rat 1**	**Rat 2**	**Rat 3**	**Rat 1**	**Rat 2**	**Rat 3**	
CA1	2	0	26	2	5	34	2	2	18	91
CA3	22	4	11	8	10	11	9	17	8	100
Total	24	4	37	10	15	45	11	19	26	191

It appeared that neuronal spikes contained slightly higher amounts of spatial information (measured in bit/spike, Figure 4C) (Skaggs et al., 1993) when MUS was injected into PER, compared to CT in both CA1 and CA3. A Two-Way ANOVA showed, however, no significant drug effect [*F*_(1, 133)_ = 3.57, *p* = 0.06] and only showed a significant effect of subfield [*F*_(1, 133)_ = 4.61, *p* < 0.05]. There was no significant interaction between the drug and subfield [*F*_(1, 133)_ = 0.27, *p* > 0.5]. The results overall suggest that the PER inactivation with MUS did not significantly alter the general firing characteristics of hippocampal pyramidal neurons.

### Inactivation of the PER disrupted object-in-place firing patterns of hippocampal neurons

It was previously shown that, after the rat learned the current task, the neuronal firing patterns in the hippocampus became similar between the trial conditions categorized as O-P trial type (Figure 1C). That is, upon encountering the two trial conditions associated with different object configurations in the choice platform in the same arm (resulting in the target object placed in opposite positions in the platform between the two trial conditions), the rat was able to choose the target object reliably (despite opposite turning responses required upon entering the choice platform) only after learning. During a pre-learning stage, however, rats tended to choose a particular object based on a response strategy (i.e., always turning to a particular side regardless of the object identity associated with the direction; Figure 1C). In a given session, the trials in which behavioral choices were made based on O-P strategy were categorized as O-P trial type and the ones in which choices were made using R-P strategy were labeled as R-P trial type (Figure 1C). We showed in our prior studies that the firing patterns of CA1 neurons (and mPFC) were more similar in O-P trial type than in R-P trial type after, but not before, learning occurred (Lee and Kim, 2010; Kim et al., 2011).

**Figure 5:**
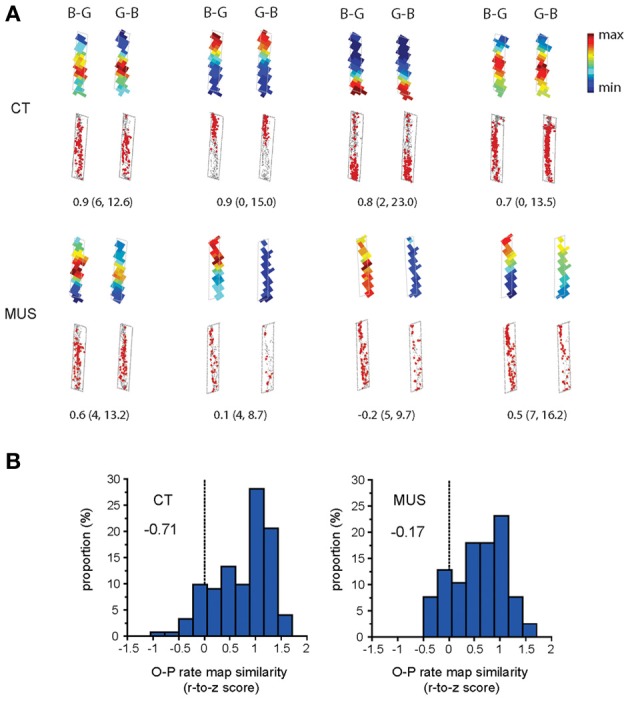
**O-P firing pattern under MUS in PER. (A)** Representative examples of the within-arm firing rate maps associated with O-P trial type. B-G and G-B indicate the configural arrangement of objects (B: obj-B, G: obj-G) in the choice platform at the end of the arm. Raw spiking maps using the same illustration scheme as Figure 4A are shown under the rate maps. Only the firing patterns for the areas included in analyses (i.e., arm regions) are shown (choice platform thus excluded). The number underneath each pair of rate maps indicates the Pearson correlation coefficient. The minimum and maximum firing rates (Hz) used for color-scaling the rate maps are parenthesized. **(B)** The neuronal distribution of the Pearson correlation coefficients (Fisher r-to-z transformed scores) calculated between the firing rate maps associated with O-P trial type in each drug condition. A more negatively skewed distribution means higher similarity in firing patterns between different trial conditions belonging to the same strategy-dependent trial type. Note the highly negatively skewed distribution in CT compared to MUS (skewness measures provided inside the graphs) for rule-compatible (O-P) trial type.

We examined whether the inactivation in PER disrupted the O-P firing pattern in the hippocampus. The amount of O-P firing pattern was measured by calculating a Pearson correlation coefficient between the rate maps associated with the trial conditions belonging to the same trial type (i.e., the two trial conditions in a certain arm with the arm-specific target object on the left side and the right side in the choice platform; Figure 1C). In the CT condition, cells exhibited robust O-P firing patterns, showing very similar firing rate maps between the trial conditions belonging to the same trial type (Figure 5A). This was confirmed by a negatively skewed distribution (skewness = −0.71) of the correlation coefficients between rate maps associated with O-P trial type in CT (Figure 5B). In contrast, the O-P firing pattern was significantly disrupted in the MUS condition (Figure 5A), and this resulted in a less negatively skewed distribution (skewness = −0.17) of the correlation coefficients between the firing rate maps (Figure 5B) as compared to the CT (*D* = 0.25, *p* < 0.05, Kolmogorov–Smirnov test). The results suggest that the inactivation of the PER significantly disrupted the hippocampal neuronal capability of emitting similar firing patterns between the trial conditions that needed to be processed in association with O-P strategy.

### Inactivation of the PER affected the object-in-place firing in CA1, but not in CA3

**Figure 6:**
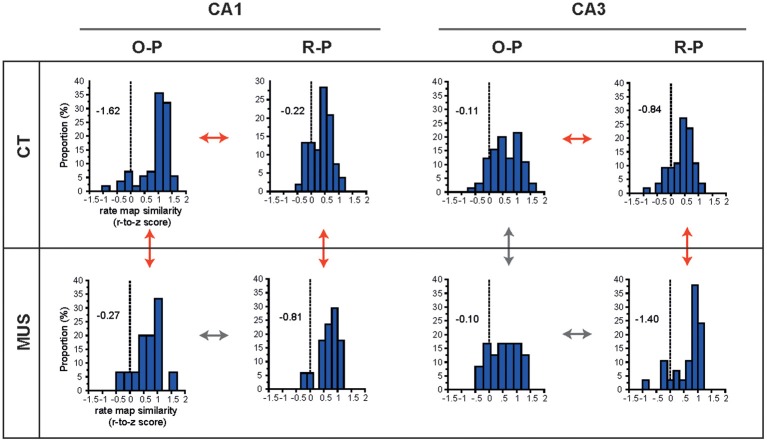
**O-P and R-P firing patterns in hippocampal subfields**. The distributions of r-to-z transformed scores of correlation coefficients between firing rate maps for different trial types (i.e., O-P and R-P) are presented for the combinatorial conditions between subfield and drug injection. Skewness measures are provided inside the graphs. Red arrows and gray arrows indicate significant and insignificant differences, respectively, between the selective pairs of distributions.

We showed previously in CA1 that the rate-map similarity was high between R-P trial conditions when the rat showed response bias prior to learning, but the R-P rate-map similarity significantly decreased as learning occurred (and as the rate-map similarity in O-P trials increased; Lee and Kim, 2010; Kim et al., 2011). In order to test whether PER-MUS differentially affected the strategy-dependent firing patterns in CA1 and CA3, we performed a full examination of the rate-map similarities between O-P trial conditions and between R-P trial conditions for the hippocampal subfields CA1 and CA3 between the drug conditions (Figure 6). In normal conditions (CT), the CA1 subfield maintained higher rate-map similarity in the O-P trial type than CA3 (compare O-P histograms in CT condition between CA1 and CA3 in Figure 6; *D* = 0.45, *p* < 0.0001, Kolmogorov–Smirnov test). This subregional difference, however, was eliminated by MUS (compare O-P histograms in MUS condition between CA1 and CA3 in Figure 6; *D* = 0.27, *p* > 0.5). This was mainly attributable to the degraded O-P rate-map similarity in CA1 (red arrow for comparing CA1 O-P histograms between CT and MUS in Figure 6; *D* = 0.47, *p* < 0.01), but not in CA3 (gray arrow for comparing CA3 O-P histograms between CT and MUS in Figure 6; *D* = 0.11, *p* > 0.5), after MUS injections in PER.

In contrast to the dissociation between CA1 and CA3 with respect to the O-P rate-map similarity under MUS, the R-P rate-map similarity increased in both CA1 and CA3 as rats made more errors in MUS conditions (red arrows for comparing R-P histograms between CT and MUS in CA1 and CA3 in Figure 6; *D* = 0.48, *p* < 0.01 for CA1 and *D* = 0.49, *p* < 0.001 for CA3, Kolmogorov–Smirnov tests). In normal conditions (CT), the rate-map similarity in O-P trial type was significantly higher than the R-P rate-map similarity (red arrows for comparing O-P and R-P histograms in CT) in both CA1 (*Z* = −5.55, *p* < 0.0001, Wilcoxon signed rank test) and CA3 (*Z* = −3.29, *p* < 0.001), whereas no such differences were found between O-P and R-P trial types (gray arrows for comparing O-P and R-P histograms in MUS) in both CA1 (*Z* = −0.39, *p* > 0.5) and CA3 (*Z* = −1.68, *p* = 0.09) once PER was inactivated by MUS.

In sum, we verified our previous findings (in CA1) that, in both CA1 and CA3 in normal conditions, the firing patterns between different O-P trial conditions were more similar to each other than the R-P firing patterns when PER functioned normally. A novel finding is that O-P strategy-compatible firing patterns in normal conditions were more prominent in CA1 than in CA3 although both subfields displayed significant effects of O-P strategy. When the PER was inactivated, however, the rate-map similarity in R-P trial type significantly increased in both CA1 and CA3. Interestingly, the increases in rate-map similarity in R-P trial type in CA1 and CA3 occurred at the expense of significant decrease in rate-map similarity in O-P trial type in CA1, but not in CA3 when MUS was injected into PER. It appears that, when PER was inactivated, CA3 showed a “bistability” for increasing the rate-map similarity for R-P trial type while maintaining the O-P rate-map similarity. Such bistability was not observed in CA1 as the network state in CA1 broke down for O-P firing similarity to increase R-P firing similarity under MUS in PER. The results overall suggest that the similar firing patterns of CA1 cells that were demonstrated in the rate maps associated with the O-P trial type in the current task critically depended on the normal functions of the PER, whereas CA3 cells were relatively independent of the PER in showing such firing properties.

## Discussion

The current study shows the physiological effects of PER inactivation on hippocampal neuronal firing for the first time to our knowledge. The task demand of the current behavioral paradigm required rats to process object-place paired associative information strategically in a goal-directed fashion. That is, despite the physical differences between the two trial conditions of a particular O-P trial type (e.g., obj-B on the left food well and obj-G on the right food well in the choice platform *vs.* the opposite configural arrangement of the same two objects across different trials in a given arm; Figure 1C), the rat was required to treat the events in the same way by choosing the same target object in a certain place (i.e., arm) for obtaining reward. As shown in the current study and in our previous studies (Lee and Solivan, 2008, 2010; Jo and Lee, 2010a,b), inactivations in PER as well as the dorsal hippocampus almost completely abolished the animal's capability of making proper behavioral choices according to such task demands. Our results from the current study suggest that MUS mostly damaged the O-P strategy-dependent firing patterns of CA1 neurons, leaving such firing patterns in CA3 relatively unaffected in comparison to R-P strategy-dependent firing patterns in both regions. We cannot rule out, however, that the amount of rate-map similarity in CA3 might not be dependent on learning the O-P strategy and further studies for recording CA3 throughout learning stages are needed to investigate this issue.

Our study suggests that the inactivations in PER qualitatively influence specific information processing in the hippocampus, leaving the overall firing rates in CA1 and CA3 neurons mostly unaffected. This may be attributable to the fact that a large proportion of PER efferents reaches the hippocampus indirectly by way of the entorhinal cortex (mostly via LEC; Witter and Amaral, 2004; Kerr et al., 2007). Interestingly, the amount of spatial information contained in spiking activity showed a trend of increase (*p* = 0.06) in the hippocampus with MUS in PER in our study (Figure 4D). This could reflect the inhibition of non-spatial information indirectly and directly from PER to the hippocampus, and perhaps a resulting increase in spatial information fed from a different route (i.e., POR-MEC stream). The results are in line with prior studies that have shown minimal spatial firing characteristics of PER and LEC neurons compared to MEC neurons (Hargreaves et al., 2005; Knierim et al., 2006; Yoganarasimha et al., 2010; Deshmukh and Knierim, 2011; Deshmukh et al., 2012). It is likely that the hippocampal cells could maintain (or enhance) spatial firing under MUS in PER because the MEC-driven spatial information was fed normally (or even boosted) to the hippocampus (Hargreaves et al., 2005). Our physiological results thus predict that a behavioral task that requires the animal to contextually (including spatial locations) respond to objects should require the PER in addition to the hippocampus (Gaffan and Parker, 1996; Gaffan et al., 2000; Bussey et al., 2001; Jo and Lee, 2010a,b).

It is speculated that the stronger O-P firing patterns in CA1 than in CA3 in normal conditions (CT, Figure 6) may be the result of testing the well-trained rats in the current study. Specifically, the literature on hippocampal subregional functions shows that CA3 is critical for the acquisition of new memory or when changes are induced in the environment, presumably involving NMDA receptor-dependent plasticity, whereas CA1 is important for retrieving old memories (Lee and Kesner, 2002, 2003, 2004; Nakazawa et al., 2003; Lee et al., 2005; Cravens et al., 2006; Rajji et al., 2006; McHugh and Tonegawa, 2009). The rats in our study were overtrained and showed asymptotic performance by the time they were tested under the drug-injection conditions. At this stage, it is possible that CA3 was no longer engaged as much as CA1 in this task unless novel object-place paired associations needed to be learned. It may be also why the PER inactivation affected the O-P rate-map similarity in CA1 only in this study. It needs to be determined in the future whether PER-MUS would affect CA3 if MUS were injected during the acquisition phase of an object-place paired-associate task.

It is worth mentioning that, with the constraint of targeting both CA1 and CA3 in the same animal with a single electrode bundle containing multiple tetrodes, most of our CA1 recording was made in the proximal CA1 region (Figure 2A). This was largely because we intentionally targeted the CA3a-b region, and wanted to record the CA1 region (i.e., proximal CA1) connected to the CA3a-b area. We have targeted CA3a-b because that is the region where relatively rich recurrent collateral networks exist within CA3 (Ishizuka et al., 1990). Recurrent collaterals are critical for a computational process known as pattern completion or generalization (O'Reilly and McClelland, 1994; Lee et al., 2004b; Vazdarjanova and Guzowski, 2004) and we originally reasoned that pattern completion might be necessary for treating the two different object configurations associated with an arm similarly by emitting the same response to one of the objects according to O-P strategy (Figure 1C). This was not the case because, although there were still significant differences in the firing patterns between O-P and R-P trial types in CA3 in CT (Figure 6), the CA3 network was less affected than CA1 by the relevant rule or strategy (see the less skewed O-P distribution of CA3 in CT than the O-P distribution of CA1 under the same drug condition in Figure 6). What is also interesting is that the CA3 network behavior in O-P trial type was more immune to MUS injected into PER while there was still a significant increase in the R-P trial type-compatible firing under PER-MUS (more negatively skewed R-P rate-map similarity distribution under MUS than with CT). In contrast, the highly skewed distribution of the O-P rate-map similarity in CA1 in CT broke down with MUS injection in PER, which is reminiscent of the CA1 network showing similar behavior in response to environmental changes (Lee et al., 2004b). The results confirm that CA1 is more sensitive to changes in the external environment (which was artificially induced in this study by inactivating the PER inputs to the hippocampus) than CA3. Importantly, our study confirms that this network behavior is correlated with behavioral performance in a goal-directed task.

Anatomical studies have shown that the proximal CA1 receives major inputs from MEC, whereas the distal CA1 receives its inputs mostly from LEC (Witter and Amaral, 2004; Knierim et al., 2006; Kerr et al., 2007). Given the currently dominant hypothesis positing two independent streams of PER-LEC (to distal CA1) and POR-MEC (to proximal CA1) carrying non-spatial and spatial information, respectively, it is interesting that PER-MUS affected the neural firing patterns of proximal CA1 neurons in our study. Although this needs further investigations (e.g., recording both proximal and distal regions of CA1 while inactivating PER), it suggests a possibility that spatial vs. non-spatial information streams may not be segregated in CA1 as clearly as implicated in the literature. Dynamic feedforward and feedback connections among PER, POR, LEC, and MEC (Witter and Amaral, 2004; Knierim et al., 2006; Kerr et al., 2007) as well as among the subfields of the hippocampus may have contributed to our results.

It has been shown that disrupting PER-hippocampal connections or PER lesions fail to impair simple object discrimination, but the same manipulation severely disrupts object-place paired associate memory (Bussey et al., 2001; Jo and Lee, 2010a,b). This may be related to the PER functions in resolving ambiguity in object recognition (Bussey et al., 2002) when the same objects need to be recognized against different backgrounds (perhaps almost perceived as a complex scene by an animal approaching them from a distance) as the objects switched their relative positions (e.g., arms) in the same environment across trials. In fact, when different sets of objects were used for the two arms in a study similar to the one used in the current study, PER was not required (Jo and Lee, 2010a). In addition, perturbations in PER indeed produced deficits in discriminating feature-overlapping visual stimuli when scene-like complex visual stimuli were used (Bussey et al., 2002; Winters et al., 2010) but not when relatively simple visual stimuli needed to be discriminated (Clark et al., 2011). Further investigations are needed to delineate the conditions in which PER is engaged, but our results strongly suggest that the PER functions can be ideally tested in a task in which “what” information needs to be considered in conjunction with “where” information. This arguably is a more natural way of experiencing an event and remembering it than processing either type of information alone, and it appears that the hippocampal-PER interaction seems critical when such contextual object recognition is necessary (Lee and Lee, 2013).

### Conflict of interest statement

The authors declare that the research was conducted in the absence of any commercial or financial relationships that could be construed as a potential conflict of interest.
